# Synergistic effect of *Lysinibacillus sphaericus* and glyphosate on temephos-resistant larvae of *Aedes aegypti*

**DOI:** 10.1186/s13071-020-3928-3

**Published:** 2020-02-12

**Authors:** Laura Bernal, Jenny Dussán

**Affiliations:** Microbiological Research Center (CIMIC), Department of Biological Sciences, Universidad de Los Andes, Carrera 1 No. 18 A-12, Bogotá, 111711 Colombia

**Keywords:** *Lysinibacillus sphaericus*, *Aedes aegypti*, Glyphosate, Synergy

## Abstract

**Background:**

Glyphosate-based herbicides are one of the most commonly used compounds to control perennial weeds around the world. This compound is very persistent in the environment and tends to filter into aquatic ecosystems, affecting non-target species such as mosquito larvae. *Aedes aegypti* mosquitoes are vectors of multiple arboviruses such as dengue and Zika. Glyphosate can be degraded into non-harmful environmental compounds by *Lysinibacillus sphaericus*, a spore forming bacterium which can also kill *Ae. aegypti* larvae. In this study, we assessed the effect of glyphosate concentrations, typically used in Colombia, on the entomopathogenic activity of *L. sphaericus* against *Ae. aegypti* larvae.

**Methods:**

Bioassays and toxicity curves were performed to compare the larval mortality between different treatments with and without bacteria and glyphosate (Roundup 747®). Larvae were exposed to both bacteria and glyphosate by adding the compound on chloride-free water. Comparisons were made using both probit regression and ANOVA analysis.

**Results:**

ANOVA showed a significant difference in larval mortality when adding glyphosate and *L. sphaericus* at the same time. Thus, a positive synergic effect on larval mortality was found when *L. sphaericus* and glyphosate were mixed. According to probit analysis, median lethal dose (LD50) for bacterial mixture was of 10^6.23^ UFC/ml and for glyphosate was 2.34 g/l.

**Conclusions:**

A positive synergic effect on the mortality of larval *Ae. aegypti* when exposed to *L. sphaericus* mixture and glyphosate was found. Molecular studies focusing on the toxin production of *L. sphaericus* are required to understand more about this synergistic effect.
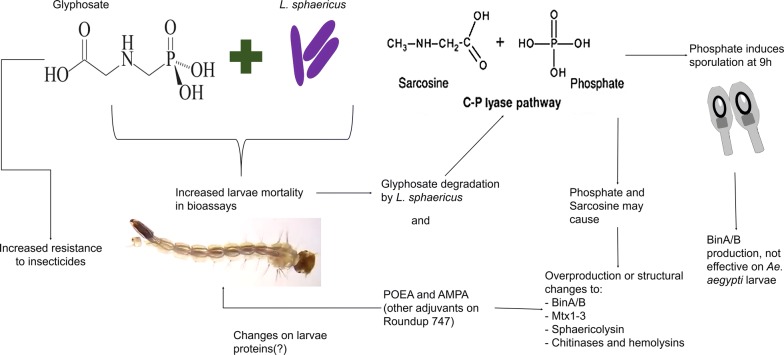

## Introduction

Glyphosate-based herbicides are one of the most commonly used compounds around the world to control perennial weeds [1]. Traces of glyphosate, and its main metabolite aminomethylphosphonic acid (AMPA), have been found in the drinking water and human urine of farmers in Mexico [2], in water from soybean crops in Argentina and water samples of Mideast USA [3, 4]. Glyphosate and AMPA are persistent in the environment and are toxic to non-targeted organisms including mosquito larvae [5].

In Colombia, glyphosate is used not only for agriculture but also for massive aerial aspersions to eliminate the illicit coca crops [6]. Since 1999, the “Plan Colombia” policy increased the ratio of fumigation events which not only failed to control the amount of coca crops, but also endangered other non-target species in the process [6–9]. The contamination of water ecosystems by glyphosate and AMPA are events of great environmental concern in Colombia.

Biodegradation of glyphosate on the environment is reported to take place by two metabolic pathways: C-N oxidase and C-P lyase [10]. The C-N oxidase pathway produces AMPA and glyoxylate and is a common pathway for the mineralization of glyphosate in both soils and water ecosystems. On the other hand, the C-P lyase pathway breaks down the C-P bond producing orthophosphate ion and glycine, which are non-toxic compounds [11].

Furthermore, little is known about the interaction of glyphosate with organisms other than plants. Some studies demonstrated that glyphosate had an impact on small populations of bees and mosquito larvae, making them more susceptible to neurological damage and resistant to insecticides [12, 13]. These findings suggest that further investigation is needed to establish the effect of glyphosate and AMPA on non-target species that have more significant roles in trophic chains and public health.

In Colombia, *Aedes aegypti* is the principal vector of multiple arboviruses such as dengue, Zika and chikungunya [14]. These mosquitoes are very important vectors due to their diurnal activity and preference for human blood. *Aedes aegypti* larvae are found in water in both domestic and peridomestic environments, and the mosquito resistance to insecticides seems to be increasing [15]. La Mesa-Cundinamarca is a well-known zone in Colombia due to its temephos-resistant mosquitoes. This compound is commonly used as a control method for *Ae. aegypti* larvae even though more than 70% of *Ae. aegypti* populations in Colombia are resistant to it [16]. Other insecticides may be toxic or cause secondary effects on non-target organisms. Hence, it is crucial to search for complementary control interventions such as biological agents that are environmentally friendly.

Many microorganisms are used as biological agents against plagues; for example, the mycoparasite fungus *Trichoderma harzianum* is used against phytopathogenic species, and the entomopathogenic bacterium *Bacillus thuringiensis* is applied to crops [17, 18]. Recently, Colombian strains of *Lysinibacillus sphaericus*, a well-known entomopathogenic gram-positive innocuous bacterium, have been shown to demonstrate strong activity against *Culex*, *Aedes* and *Anopheles* larvae [19, 20]. *Lysinibacillus sphaericus* is not only a very effective biocontrol agent for mosquito larvae, but it is also capable of removing toxic metals from water, promoting plant growth, and metabolizing glyphosate by a pathway that does not produce AMPA but glycine and orthophosphate ion instead [21–25].

In this study, we examined the effect of glyphosate concentrations typically used in Colombia on the entomopathogenic activity of *L. sphaericus* against temephos-resistant *Ae. aegypti* larvae.

## Methods

### *Lysinibacillus sphaericus* strains

The strains of *L. sphaericus* used in this study were the WHO reference strain 2362 and *L. sphaericus* III(3)7, a Colombian strain isolated from a native oak tree (*Quercus humboldtii*) [26]. This mixture was previously shown to be the most lethal for *Ae. aegypti* larvae [27].

### *Aedes aegypti* maintenance

*Aedes aegypti* third-instar larvae were collected from La Mesa Cundinamarca (4°38′05.9″N, 74°27′45.4″W), a well-known area for temephos-resistant mosquitoes. Larvae were kept at 28 ± 0.03 °C and a relative humidity of 70% under 12:12 h light/dark photoperiod. The experiments were initiated 24 h after collecting the larvae.

### Formulation of test agents and synergistical bioassay conditions

*Lysinibacillus sphaericus* strains were grown in nutritive agar (CM0003; Oxoid, Thermo Fisher Scientific, Hampshire, UK) for 15 h at 30 °C. Cells were collected and resuspended in 10 ml of distilled sterile water followed by a series of dilutions to set the initial inoculum according to the concentrations established. To determine the median lethal dose 50 (LD50_2362+III(3)7_) of the bacterial mixture on the larvae, a toxicity curve was performed. In total, five different concentrations of bacterial inoculum were used: 10^5^ UFC/ml; 10^6^ UFC/ml; 10^7^ UFC/ml; 10^8^ UFC/ml; and 10^9^ UFC/ml.

Monsanto’s glyphosate formulation Roundup 747® was used as the only source of glyphosate. To determine the median lethal dose 50 (LD50_gly_) of the larvae to glyphosate exposure, five different concentrations were used: 0.5 g/l; 1.0 g/l; 1.69 g/l; 2.0 g/l; and 2.5 g/l. LD50 probit analysis was applied to both in order to establish the concentrations to conduct the bioassays (bioassay treatments are described in Table 1).

**Table 1:** Description of treatments implemented in the study

Treatment	*Ae. aegypti* larvae	*L. sphaericus* (2362 +III(3)7)	Glyphosate (1.69 g/l)
Control	+	−	−
Larvae + (2362 + III(3)7)	+	+	−
Larvae *+* glyphosate	+	−	+
Larvae *+* 2362 + III(3)*7* *+* glyphosate	+	+	+

*Key*: +, added; -, not added

*Note: Lysinibacillus sphaericus* strains were 2362 and III(3)7

The bioassays and LD50 were set-up following the procedure described by Rojas and Dussán [19]: 20 *Ae. aegypti* third-instar larvae were placed into glass flasks (7.1 × 7.1 × 7.8 cm) containing 30 ml of chloride-free tap water. Additionally, 300 µl of bacterial suspension was added to reach the final concentration in each test. Similarly, glyphosate was added until the final concentration was reached. Larvae were maintained at 28 ± 0.3 °C, a relative humidity of 70% and a 12/12 h light/ dark photoperiod. Mortality was reported after 24 h and 48 h of exposure; larvae with no response to physical stimuli or unable to attach to the surface were counted as dead. Each experiment was tested in triplicate, and all bioassays and LD50 determinations were replicated.

Finally, an aliquot of water at 0 h, 24 h, 48 h was taken in order to perform plating assays and confirm bacteria behaviour after addition in every treatment. Plating was performed on nutrient agar, incubating for 12 h at 30 °C.

### Statistical analysis

All statistical tests were carried out using the R 3.1.2 statistical package [28] and a significance level of *P* < 0.05 was chosen for every test. To determinate the difference in larval mortality between bioassays, ANOVA tests were performed.

## Results and discussion

LD50 for the mixture of *L. sphaericus* 2362 and III(3)7 was found to be 10^6.23^ UFC/ml (*R*^2^ = 0.9934; y = 12.829x − 18.566). According to these results and previous studies, we decided to use a constant concentration of 10^7^ UFC/ml [27]. Given these results, all bioassays were also calibrated to that concentration. After the measurements of larval mortality, we observed that the LD50_gly_ was 2.34 g/l (*R*^2^ = 0.947; y = 3.452x + 1.6483). This result allows us to use field concentrations of glyphosate (1.69 g/l). This concentration was established based on the way Colombian farmers prepare glyphosate for usage, in which an entire contents of Roundup 747® package is dissolved in 20 l of water.

Furthermore, as far as we know, the maximum concentration tested on *Ae. aegypti* was 0.2 g/l [29], which is 10 times less than the concentration used in this study. With this information, we can assume that the resistance of the larvae from La Mesa-Cundinamarca to glyphosate is due to their exposure to higher concentrations of glyphosate and other organophosphate compounds such as temephos or other insecticides. Temephos is not only relevant for this study, but also reflects the real exposure of non-target species to the indiscriminate use of both insecticides and glyphosate in rural Colombia.

**Fig. 1:**
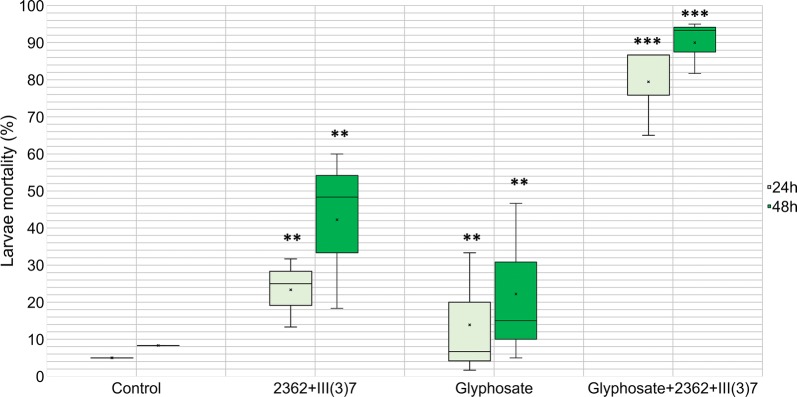
Larval mortality for the different treatment assays. Boxes represent quartile range, crosses inside the plot represent the media of the assays (average larval mortality in bacterial mixture at 24 h, 23.3%; average mortality in glyphosate at 24 h, 20%; average mortality in glyphosate and bacterial mixture at 24 h, 79.44%) Significant differences between Glyphosate+2362+III(3)7 and the other treatments on the same time of larval mortality 24 and 48 h was found (***P < 0.00001). Additionally, there were significant differences between the control and the treatments with only bacteria or glyphosate (**P < 0.007). No significant differences were found between the treatments with only bacteria or only glyphosate

A significant difference was observed in the mortality of *Ae. aegypti* larvae exposed to glyphosate and the bacterial mixture compared to the treatments and control at the same measurement time (Fig. 1). At 24 h, the larval mortality in the bacterial mixture with glyphosate was 4-fold higher that what was observed in the other treatments (ANOVA: *F*_(3, 44)_ = 67.87, *P* < 0.0001; average larval mortality in the bacterial mixture: 23.3%; average larval mortality in glyphosate: 20%; and average larval mortality in the glyphosate and bacterial mixture: 79.44%).

There were significant differences in larval mortality at 48 h between treatment and control experiments, when compared to *L. sphaericus* and glyphosate experiments (ANOVA: *F*_(3, 44)_ = 47.37, *P* < 0.0001; average larval mortality in the bacterial mixture: 42.2%; average larval mortality in glyphosate: 22.2%; and average larval mortality in the glyphosate and bacterial mixture: 90%).

**Fig. 2:**
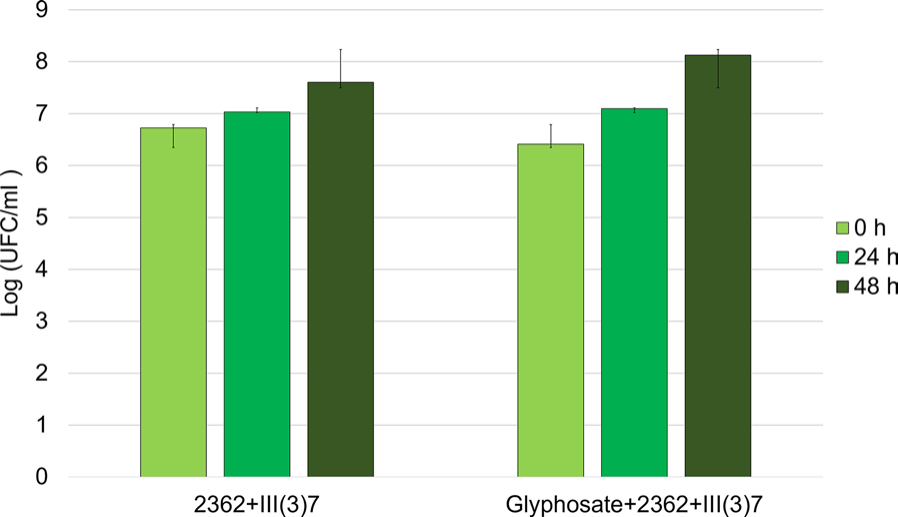
Plating assays of the *L. sphaericus* mixture on the different treatments at three time points 0, 24, 48 h, post-addition of glyphosate or bacteria (1 ml aliquots of water from the bioassays were used to perform this titration)

These results indicate that the addition of glyphosate to *L. sphaericus* can produce a synergic effect on larvicidal activity. To verify whether the *L. sphaericus* titer was affected by glyphosate (Roundup 747®) addition, plating assays were performed at 0, 24 and 48 h after inoculation (Fig. 2). In this case, the bacterial titer did not change, yet the sporulation was faster in the assays with glyphosate.

In this study, we found a synergetic behaviour on mosquito larval mortality when glyphosate (Roundup 747®) was added to the bacterial mixture. As reported by González and Dussán [25], the sporulation was stimulated at 9 hours after the addition of glyphosate. And, as mentioned before, sporulation is stimulated by glyphosate which means that BinA/B production must have increased. If this production had any effect on larval mortality, mortality should have been visible at least at 9 to 10 hours of the setup of the bioassays, yet mortality was observed after 20 hours, which may suggest that BinA/B production may not be the main cause of larval mortality. Furthermore, these results are intriguing to observe because *Ae. aegypti* larvae are immune to toxins BinA/B produced primarily in the sporulation process of *L. sphaericus* [30]. Thus, the expected reaction would be to see a decline of larval mortality once the sporulation process of the bacteria has been completed in no more than 9 hours, yet, our results showed a different behaviour.

This evidence drives us to generate two hypotheses. The first is that in the Roundup 747® formulation, there is a particularly toxic surfactant used to increase glyphosate function, polyethoxylated tallow amine (POEA) [31]. Many reports affirm that this compound induces DNA damage in zebra fish (*Danio rerio*) embryos and is lethal to all aquatic species of bacteria, algae and amphibians [31, 32]. In our study, given the average mortality presented in the treatments where glyphosate alone was used (Fig. 1), we conclude that larval mortality can be partly assigned to the adjuvants of the Roundup 747® formulation. However, there may be an interaction between the adjuvants and *L. sphaericus* that dramatically increases toxicity to the mosquito larvae. More in-depth studies are required to prove this.

Our second hypothesis is that, given the evidence presented above, *L. sphaericus* has the capability of degrading glyphosate into two main molecules, glycine and orthophosphate [22], which can both be easily used by the larvae and the bacteria in different metabolic pathways such as phosphorylation of proteins. Increasing the overexpression of different proteins such as the Mtx1-3, chitinase, and the S layer protein also increases the toxicity against *Ae. aegypti* larvae, which may explain the difference between the mortality rates when *L. sphaericus* and glyphosate are used alone or mixed [33].

To test which molecules have the greatest effect on larvicide activity of the toxins produced by *L. sphaericus*, bioassays with *L. sphaericus* strains that are not able to produce BinA/B toxins can help to understand if synergy in larval mortality is explained by overproduction of said toxins or whether it is due to other proteins such as Mtx1-3, sphaericolysin or bacteria chitinases [20, 34]. On the other hand, it is necessary to test different compounds and chemical species produced by glyphosate degradation by *L. sphaericus*, in particular glycine and orthophosphates.

The toxicity can be mediated by different phenomena such as overproduction or changes in the tertiary structure of the proteins as reported for the Mtx3 protein in which small changes to its tertiary structure drastically augmented its toxicity [20]. Also, Nishiwaki et al. [34] found that the sphaerycolisin on *L. sphaericus* A3-2 (a non-toxic strain) augmented the toxicity mediated by cholesterol-dependent cytolysins.

## Conclusions

A positive synergic effect on the mortality of larval *Ae. aegypti* when exposed to *L. sphaericus* mixture and glyphosate was found. The levels of glyphosate, AMPA, and glycine need to be measured in order to clarify whether the larval mortality is mediated by the metabolism of glyphosate by *L. sphaericus* or by the effect of the adjuvant agents on the metabolism of both the larvae and the bacteria. Studies on the production of *L. sphaericus* toxins to determine the effect of glyphosate, glycine and orthophosphate on bacterial metabolism are required. Also, measurements on the production of bacterial toxins when exposed to the POEA adjuvant present in the Roundup 747® formulation will help gain a better comprehension of this synergy effect. Finally, larval tolerance to glyphosate is as concerning as it is intriguing, given the ability of *L. sphaericus* to control those populations of mosquitoes that are so resistant to different toxic compounds. These results clearly show the need to study the present conditions of these vectors as well as the implementation of mosquito management plans that avoid the use of toxic compounds.

## Data Availability

The data generated and the material used during the present study are available from the corresponding author upon reasonable request.

## Abbreviations

Bin A/B: binary toxin
AMPA: aminomethylphosphonic acid
LD50: amount of toxic agent (bacteria or glyphosate) at which 50% of the population dies

## References

[1] Xu J, Smith S, Smith G, Wang W, Li Y (2019). Glyphosate contamination in grains and foods: an overview. Food Control..

[2] Rendón J, Dzul-caamal R (2017). Glyphosate residues in groundwater, drinking water and urine of subsistence farmers from intensive agriculture localities: a survey in Hopelchén, Campeche, Mexico. Int J Environ Res Public Health..

[3] Peruzzo PJ, Porta AA, Ronco AE (2008). Levels of glyphosate in surface waters, sediments and soils associated with direct sowing soybean cultivation in north pampasic region of Argentina. Environ Pollut..

[4] Battaglin WA, Kolpin DW, Scribner EA, Kuivila KM, Sandstrom MW, William A (2005). Glyphosate, other herbicides, and transformation products in Midwestern streams. J Am Water Resour Assoc..

[5] Sikorski Ł, Baciak M, Bęś A, Adomas B (2019). The effects of glyphosate-based herbicide formulations on *Lemna minor*, a non-target species. Aquat Toxicol..

[6] Rincón-ruiz A, Kallis G (2013). Caught in the middle, Colombia’s war on drugs and its effects on forest and people. Geoforum..

[7] Camacho A, Mejía D (2017). The health consequences of aerial spraying illicit crops: the case of Colombia. J Health Econ..

[8] Arias V, Rodriguez A, Bardos P, Naidu R (2018). Contaminated land in Colombia: a critical review of current status and future approach for the management of contaminated sites. Sci Total Environ..

[9] Sihtmäe M, Blinova I, Künnis-Beres K, Kanarbik L, Heinlaan M, Kahru A (2013). Ecotoxicological effects of different glyphosate formulations. Appl Soil Ecol..

[10] Cecilia D, Maggi F (2018). Analysis of glyphosate degradation in a soil microcosm. Environ Pollut..

[11] Sviridov AV, Shushkova TV, Ermakova IT, Ivanova EV, Epiktetov DO, Leontievsky AA (2015). Microbial degradation of glyphosate herbicides (review). Prikl Biokhim Mikrobiol..

[12] Herbert LT, Vázquez DE, Arenas A, Farina WM (2014). Effects of field-realistic doses of glyphosate on honeybee appetitive behaviour. J Exp Biol..

[13] Riaz MA, Poupardin R, Reynaud S, Strode C, Ranson H, David JP (2009). Impact of glyphosate and benzo[a]pyrene on the tolerance of mosquito larvae to chemical insecticides. Role of detoxification genes in response to xenobiotics. Aquat Toxicol..

[14] Leta S, Jibat T, De Clercq EM, Amenu K, Kraemer MUG, Revie CW (2018). Global risk mapping for major diseases transmitted by *Aedes aegypti* and *Aedes albopictus*. Int J Infect Dis..

[15] Aponte A, Penilla RP, Rodríguez AD, Ocampo CB (2019). Mechanisms of pyrethroid resistance in *Aedes* (*Stegomyia*) *aegypti* from Colombia. Acta Trop..

[16] Santacoloma L, Chaves B, Brochero HL (2012). Estado de la susceptibilidad de poblaciones naturales del vector del dengue a insecticidas en trece localidades de Colombia. Biomédica..

[17] Adnan M, Islam W, Shabbir A, Ali K, Ghramh HA, Huang Z (2019). Microbial pathogenesis plant defense against fungal pathogens by antagonistic fungi with *Trichoderma* in focus. Microb Pathog..

[18] Betz FS, Hammond BG, Fuchs RL (2000). Safety and advantages of *Bacillus thuringiensis* - protected plants to control insect pests. Regul Toxicol Pharmacol..

[19] Rojas-Pinzón PA, Dussán J (2017). Efficacy of the vegetative cells of *Lysinibacillus sphaericus* for biological control of insecticide-resistant *Aedes aegypti*. Parasit Vectors..

[20] Silva Filha MHNL, Berry C, Regis L (2014). Chapter Three. *Lysinibacillus sphaericus*: toxins and mode of action, applications for mosquito control and resistance management. Insect midgut and insecticidal proteins. Advance insect physiology.

[21] Vargas J, Dussán J (2016). Adsorption of toxic metals and control of mosquitos-borne disease by *Lysinibacillus sphaericus*: dual benefits for health and environment. Biomed Environ Sci..

[22] Naureen Z, Rehman NU, Hussain H, Hussain J, Gilani SA, Al Housni SK (2017). Exploring the potentials of *Lysinibacillus sphaericus* ZA9 for plant growth promotion and biocontrol activities against phytopathogenic fungi. Front Microbiol..

[23] Vega-Páez J, Rivas RE, Dussán J (2019). High efficiency mercury sorption by dead biomass of *Lysinibacillus sphaericus* - new insights into the treatment of contaminated water. Materials (Basel)..

[24] Pérez M, Melo C, Jim E, Dussán J (2019). Glyphosate bioremediation through the sarcosine oxidase pathway mediated by *Lysinibacillus sphaericus* in soils cultivated with potatoes. Agriculture..

[25] González-Valenzuela LE, Dussán J (2018). Molecular assessment of glyphosate-degradation pathway via sarcosine intermediate in *Lysinibacillus sphaericus*. Environ Sci Pollut Res..

[26] Dussán J, Linares D, Lozano L, Vanegas P (2006). Physiologic and genetic characterization of *Bacillus sphaericus* native strains. Rev Colomb Biotecnol..

[27] Rojas-Pinzón PA, Silva-Fernández JJ, Dussán J (2018). Laboratory and simulated-field bioassays for assessing mixed cultures of *Lysinibacillus sphaericus* against *Aedes aegypti* (Diptera: Culicidae) larvae resistant to temephos. Appl Entomol Zool..

[28] R Development Core Team. R: a language and environment for statistical computing. Vienna: R Foundation for Statistical Computing; 2015. https://www.rproject.org/. Accessed 10 Feb 2019.

[29] Bara JJ, Montgomery A, Muturi EJ (2014). Sublethal effects of atrazine and glyphosate on life history traits of *Aedes aegypti* and *Aedes albopictus* (Diptera: Culicidae). Parasitol Res..

[30] Berry C (2012). The bacterium, *Lysinibacillus sphaericus*, as an insect pathogen. J Invertebr Pathol..

[31] Tsui MTK, Chu LM (2003). Aquatic toxicity of glyphosate-based formulations: comparison between different organisms and the effects of environmental factors. Chemosphere..

[32] Brito L, Gonçalves G, Lundgren E, Rafael L, De Oliveira R, Morais D (2019). Impact of the glyphosate-based commercial herbicide, its components and its metabolite AMPA on non-target aquatic organisms. Mutat Res Gen Tox En..

[33] Rojas-Pinzón PA, Dussán J (2017). Contribution of *Lysinibacillus sphaericus* hemolysin and chitin- binding protein in entomopathogenic activity against insecticide resistant *Aedes aegypti*. World J Microbiol Biotechnol..

[34] Nishiwaki H, Nakashima K, Ishida C, Kawamura T, Matsuda K (2007). Cloning, functional characterization, and mode of action of a novel insecticidal pore-forming toxin, sphaericolysin, produced by *Bacillus sphaericus*. Appl Environ Microbiol..

